# The shape of the iceberg: quantification of submicroscopic *Plasmodium falciparum* and *Plasmodium vivax* parasitaemia and gametocytaemia in five low endemic settings in Ethiopia

**DOI:** 10.1186/s12936-017-1749-4

**Published:** 2017-03-03

**Authors:** Fitsum G. Tadesse, Lotus van den Hoogen, Kjerstin Lanke, Jodie Schildkraut, Kevin Tetteh, Abraham Aseffa, Hassen Mamo, Robert Sauerwein, Ingrid Felger, Chris Drakeley, Endalamaw Gadissa, Teun Bousema

**Affiliations:** 1 Department of Medical Microbiology, Radboud University Medical Center, Nijmegen, The Netherlands; 2 Medical Biotechnology Unit, Institute of Biotechnology, Addis Ababa University, Addis Ababa, Ethiopia; 3 Armauer Hansen Research Institute (AHRI), Addis Ababa, Ethiopia; 4 Department of Immunology and Infection, London School of Hygiene & Tropical Medicine, London, UK; 5 Department of Microbial, Cellular and Molecular Biology, College of Natural Sciences, Addis Ababa University, Addis Ababa, Ethiopia; 6 Swiss Tropical and Public Health Institute, Basel, Switzerland; 7 University of Basel, Basel, Switzerland

**Keywords:** Asymptomatic, Submicroscopic, Transmission, PCR, Serology, School, El Ǹino, Elimination

## Abstract

**Background:**

The widespread presence of low-density asymptomatic infections with concurrent gametocytes may be a stumbling block for malaria elimination. This study investigated the asymptomatic reservoir of *Plasmodium falciparum* and *Plasmodium vivax* infections in schoolchildren from five settings in northwest Ethiopia.

**Methods:**

Two cross-sectional surveys were conducted in June and November 2015, enrolling 551 students from five schools and 294 students from three schools, respectively. Finger prick whole blood and plasma samples were collected. The prevalence and density of *P. falciparum* and *P. vivax* parasitaemia and gametocytaemia were determined by 18S rRNA quantitative PCR (qPCR) and *pfs25* and *pvs25* reverse transcriptase qPCR. Antibodies against blood stage antigens apical membrane antigen-1 (AMA-1) and merozoite surface protein-1 (MSP-1_19_) were measured for both species.

**Results:**

Whilst only 6 infections were detected by microscopy in 881 slides (0.7%), 107 of 845 blood samples (12.7%) were parasite positive by (DNA-based) qPCR. qPCR parasite prevalence between sites and surveys ranged from 3.8 to 19.0% for *P. falciparum* and 0.0 to 9.0% for *P. vivax*. The median density of *P. falciparum* infections (n = 85) was 24.4 parasites/µL (IQR 18.0–34.0) and the median density of *P. vivax* infections (n = 28) was 16.4 parasites/µL (IQR 8.8–55.1). Gametocyte densities by (mRNA-based) qRT-PCR were strongly associated with total parasite densities for both *P. falciparum* (correlation coefficient = 0.83, p = 0.010) and *P. vivax* (correlation coefficient = 0.58, p = 0.010). Antibody titers against *P. falciparum* AMA-1 and MSP-1_19_ were higher in individuals who were *P. falciparum* parasite positive in both surveys (p < 0.001 for both comparisons).

**Discussion:**

This study adds to the available evidence on the wide-scale presence of submicroscopic parasitaemia by quantifying submicroscopic parasite densities and concurrent gametocyte densities. There was considerable heterogeneity in the occurrence of *P. falciparum* and *P. vivax* infections and serological markers of parasite exposure between the examined low endemic settings in Ethiopia.

## Background

The remarkable success that was documented in the control of malaria in the last decade and half has intensified efforts to achieve malaria elimination and brought eradication back on the table [1]. Detailed assessments of parasite carriage by conventional diagnostics alongside molecular investigation have uncovered a considerable proportion of malaria infections is undetected by microscopy and rapid diagnostic tests (RDT) [2, 3]. In settings where recent malaria control efforts have been successful, submicroscopic infections frequently outnumber microscopically detectable infections [3–5]. Whilst the clinical consequences of apparently asymptomatic submicroscopic infections are unknown [6, 7], infections may last for several months [8] and are associated with gametocyte production [9–11]. Gametocytes are essential for onward malaria transmission to mosquitoes. Although non-linear, the likelihood of mosquito infections increases with increasing gametocyte density [12] and microscopically detectable gametocytes are thus more likely to result in mosquito infections than submicroscopic gametocyte densities [4, 13]. Submicroscopic infections may nevertheless contribute considerably to malaria transmission because of their relative abundance in populations [10, 14].

Ethiopia has experienced a 66% decline in confirmed malaria cases between 2001 and 2011 [15]. Motivated by this decline, mainly attributed to wide-scale deployment of long-lasting insecticide-treated nets and implementation of artemisinin-based combination therapy (ACT) [15], the country set a plan to eliminate malaria in selected low-transmission settings by the end of 2020 [16]. Microscopy and RDT may be insufficiently sensitive to guide or evaluate these elimination efforts. Molecular assays to sensitively detect *Plasmodium falciparum* and *Plasmodium vivax* infections and serological assays of malaria exposure may be of great value in low-endemic settings [17, 18].

Previous studies in Ethiopia reported a high degree of submicroscopic parasite carriage [19–24] and underlined the relevance of (school) surveys using serological markers of malaria exposure to determine spatial and temporal variations [25]. However, none of these studies used serological and molecular assays together or quantified the low-density infections or circulating gametocytes. A better understanding of the distribution and contribution of submicroscopic infections to the overall parasite reservoir in low-endemic settings is a prerequisite for elimination efforts in order to shape the measures to be taken. The present study evaluated spatial and temporal variation in submicroscopic parasite and gametocyte carriage along with serological markers of malaria exposure in asymptomatic schoolchildren at five different sites in northwest Ethiopia.

## Methods

### Ethics statement

The study was reviewed and approved by the Institutional Ethics Review Board of the College of Natural Sciences at Addis Ababa University (ref. CNSDO/1/07/14), AHRI/ALERT Ethical Review Committee (ref. PO52/14) at Armauer Hansen Research Institute (AHRI), the Observational/Interventions Research Ethics Committee (ref. 8626) at London School of Hygiene and Tropical Medicine (LSHTM) and the National Research Ethics Review Committee (3.10|016\20) at the Ministry of Science and Technology of the Federal Democratic Republic of Ethiopia. Community sensitization was conducted using a cascade approach [26]. All parents of children who met the basic recruitment criteria were informed about the study and offered the choice to participate through an oral informed consent process. Written consent for the study was provided by a committee that comprised the school principal, deputy principal, classroom leaders, woreda (district) officials, elders, religious leaders and representatives of the family school association while parents maintained the right to withdraw their child from the survey.

### Study area and population

**Fig. 1:**
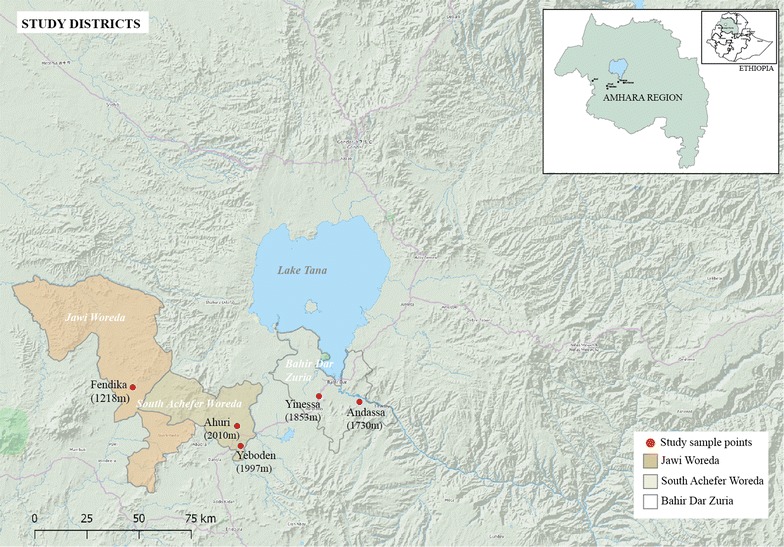
Map of study districts. The study was conducted in two surveys in the northwestern part of Ethiopia around the Lake Tana and Blue Nile river basin. Children from five elementary schools (Andassa, Yinessa, Ahuri, Yeboden and Jawi) were studied in the first survey in June 2015. During the second survey, in November 2015, students from three of the same schools (Andassa, Ahuri, Jawi) were studied

The study was conducted in five elementary schools located in the kebeles of Andassa (1730masl, N 11°30′14.5″ and E°37°29′07.9″); Yinessa (1853masl, N 11°31′42.0″ and E°37°18′26.7″), Ahuri (2010masl, N 11°24′00.7″ and E°36°56′53.0″); Yeboden (1997masl, N 11°18′50.8″ and E°36°57′49.3″) and Fendika (1218masl, N 11°34′00.3″ and E °36°29′22.9″) in the Amhara Regional State of Ethiopia (Fig. 1). Andassa and Yinessa are located in Bahir Dar Zuria woreda; Ahuri and Yeboden in Debub Achefer woreda and Fendika is the administrative town of Jawi woreda. Two seasonal peaks of malaria transmission occur in the study areas; the main peak follows the heaviest rainfall that lasts from June to September, with a smaller peak in transmission in April/May following the short rains. The study sites, except Jawi, are located in the highlands of the North-Western part of Ethiopia, around the Lake Tana and Blue Nile river basin. Most of the study sites are characterized by swampy areas or irrigation lines that are associated with small water bodies conducive for anopheline breeding.

Two cross-sectional surveys were undertaken in June and November 2015, representing the beginning of the rainy season and the peak malaria transmission season, respectively. However, due to the recent El Ǹino phenomenon that occurred during the study period the rain was intermittent and most of the areas were dry. Study participants were selected randomly among students attending the elementary schools stratified by age following protocols developed by Brooker et al. [26]. One hundred and ten students were targeted for each school from five class levels (2–6); 22 students from each class comprising 11 boys and 11 girls. The same students were invited for both surveys.

### Sample collection and microscopic parasite detection

Detailed clinical and demographic data and utilization of intervention services was recorded with a pre-tested semi-structured interview based questionnaire. Axillary body temperature was first checked for all students. If the student was found febrile (axillary temperature ≥37.5 °C) or reported a history of fever in the past 48 h, an RDT (First Response Malaria Ag (pLDH/HRP2) Combo RDT, Premier Medical Corporation Ltd., India) was immediately used to diagnose the presence of malaria parasites. If the child was not febrile, a finger-prick sample (0.3–0.4 mL blood) was collected for microscopy examination of infection with thick and thin blood smears, dried blood spots (DBS) on 3MM Whatman filter paper (Whatman, Maidstone, UK) and in an EDTA-coated microtainer tube (BD). EDTA blood samples were centrifuged immediately using portable centrifuge powered from a car battery for 3 min (at 3000 rpm) and the plasma was put into an equal volume of 0.05% sodium azide (SIGMA-ALDRICH) solution. The cell pellet was put into RNAprotect buffer (QIAGEN) in a 1:5 ratio and was used for extraction of nucleic acids to determine parasite density by qPCR and RNA-based gametocyte detection. DBS was stored in self-indicating silica gel desiccant beads (Geejay Chemicals Ltd). Plasma, cell pellet and DBS samples were shipped on ice packs from the field to the Amhara Regional Laboratory in Bahir Dar. There, samples were stored at −20 °C, before being shipped in liquid nitrogen containers to Addis Ababa and from there shipped on dry-ice to laboratories in London and Nijmegen where they were stored at −80 °C until further investigation.

Blood smears were stained at the sites for 10 min with 10% Giemsa (VWR) and then screened for malaria asexual parasites and gametocyte stages. Slides were declared negative if no parasites were observed in 100 microscopic fields. Asexual parasites and gametocytes were counted against 200 and 500 white blood cells, respectively. Slides were read independently by two microscopists in the field and at AHRI’s facility. A third, WHO certified, professional microscopist read the slides to verify the disagreement in the reading results from the two readers.

### Nucleic acid extraction, parasite and gametocyte detection by qPCR and qRT-PCR

Total nucleic acids were extracted from 600 μL aliquots containing 100 μL cell pellet plus 500 μL RNAprotect buffer (QIAGEN) using a MagNAPure LC automatic extractor and total Nucleic Acid Isolation Kit—High Performance (Roche Applied Science) and eluted in 50 μL elution buffer. qPCR for parasite detection was performed by targeting the 18S rRNA small subunit gene on DNA for *P. falciparum* and *P. vivax* using primer and probe sequences described in Hermsen et al. [27] and Wampfler et al. [28], respectively. Genomic human and parasite DNA was digested with the RQ1 DNaseI Digest Kit (Promega), then cDNA was synthesized with the High Capacity cDNA Reverse Transcription Kit (Applied Biosystems) for evaluation of transcription level of *Pfs25* and *Pvs25* to quantify *P. falciparum* and *P. vivax* gametocytes, respectively. Parasite and gametocyte quantification was done based on models developed by Wampfler et al. [28] and Koepfli et al. [11]. Briefly, plasmid constructs from Wampfler et al. [28], except the *P. falciparum* 18S rRNA target on DNA, were used to infer copy numbers, then the model was applied to calculate the corresponding parasite and gametocyte counts for *P. falciparum* and *P. vivax* parasitaemia and *P. falciparum* gametocytaemia [28] and for the *P. vivax* gametocytaemia [11]. The *P. falciparum* 18S rRNA gene was cloned into TOPO^®^-TA Cloning vector (Thermo Fisher Scientific) following the company’s protocol. Serial dilutions (10^6^, 10^5^ and 10^4^) of plasmids containing the respective amplicons in triplicates were run in parallel on every plate to generate standard curves for quantification of copy numbers. Ten percent of positive samples were retested as quality control. All cDNA samples that passed through DNA digestion were further checked for genomic DNA contamination.

The *Pfs25* sequence was amplified using GoTaq^®^ qPCR Master Mix (Promega) that contains a proprietary dye and the rest (the *Pvs25* and the *P. falciparum* and *P. vivax* 18S rRNA targets on DNA) were amplified using probe based detection using TaqMan Fast Advanced Master Mix (Applied Biosystems). Melt curves were run to evaluate detection of the specific product when the detection was not probe based. In all assays, both qPCR and qRT-PCR, 104 nM probe and 833 nM primer concentrations were used. All probes were from Life technologies (Applied Biosystems), primers were from SIGMA ALDRICH, and CFX96™ Real-Time PCR Detection System (BIO-RAD) was used.

### Serological detection of immune response to malaria antigens

Antibodies to *P. falciparum* and *P. vivax* Apical Membrane Antigen-1 (AMA-1) and Merozoite Surface Protein-1_19_ (MSP-1_19_) were detected using plasma samples that were collected and stored in equal volume of 0.05% sodium azide by Enzyme Linked Immunosorbent Assay (ELISA), as previously described [29].

### Data analysis

All analyses were performed with STATA 12 (StataCorp., TX, USA) and Graph Pad Prism 5.0 (Graph Pad Software Inc., CA, USA). Asymptomatic malaria was defined as malaria infections that lack typical clinical symptoms (absence of fever at the time of sampling or absence of history of fever 48 h before sampling) but are detectable by microscopy, rapid diagnostic test or molecular methods [30]. Antibody prevalence was determined per antigen after defining a cut-off optical density (OD) using the mixture model [31]. Prevalence was then used for the separate antigens and for the combined *P. falciparum* and the combined *P. vivax* antigens [17]. Chi square or Fisher’s Exact tests were used for dichotomous analyses; logistic or linear regression models were used for multivariate analyses. The correlation between continuous variables was assessed by Spearman correlation coefficient; continuous variables were compared between groups using the non-parametric Wilcoxon Rank Sum test.

## Results

### Parasite and gametocyte prevalence by microscopy and qPCR

During the first cross-sectional survey (June 2015), 551 students were enrolled from 5 elementary schools. The median age of the students was 12 years (IQR 11–14) (Table 1). During the second survey (November 2015), three of the five schools were revisited and 87.8% (294/335) students were successfully sampled for a second time. Microscopy detected only 6 infections (3 in each survey), which were all from Jawi. There were seven febrile students (axillary temperature ≥37.5 °C) who were all RDT negative but 1 was positive for *P. vivax* by microscopy with 6267 parasites/μL. During the second survey, 14.6% (43/294) of the children reported that they had been treated for malaria in the time-period between the two surveys (Table 1).

**Table 1:** Demographic, clinical and malariometric characteristics of study participants

	Ahuri	Andassa	Jawi	Yeboden	Yinesa	Total
Characteristics						
Female sex, % (n/N)	52.3 (57/109)	49.6 (58/117)	48.1 (51/106)	50.9 (56/110)	42.5 (45/106)	48.7 (267/551)
Age in years, median (25th–75th percentile)	13 (11–14)	12 (11–13)	12 (10–14)	12 (11–13)	12 (10–14)	12 (11–14)
Fever (temperature ≥37.5 °C)						
Survey 1, % (n/N)	0.9 (1/109)	1.7 (2/117)	0	2.8 (3/109)	0	1.1 (6/536)
Survey 2, % (n/N)	0	0	1.1 (1/94)	ND	ND	0.3 (1/294)
Reported malaria between the two surveys						
	12.2 (12/98)	6.3 (6/96)	25.0 (25/100)	ND	ND	14.6 (43/294)

Parasite prevalence by (DNA-based) qPCR during the first survey was 7.8% (43/551) for *P. falciparum* and 2.4% (13/551) for *P. vivax* (Table 2). During the second survey parasite prevalence was 12.9% (38/294) for *P. falciparum* and 4.8% (14/294) for *P. vivax*. Of all parasite-positive individuals, 7.1% (4/56) and 5.4% (3/52) had mixed *P. falciparum* and *P. vivax* infections in the first and second survey, respectively. Nine individuals (29%, 9/31) who were *P. falciparum* positive in the first survey and sampled again in the second survey were again *P. falciparum* positive; 28 (10.7%, 28/263) acquired a *P. falciparum* infection after being negative in the first survey. Two individuals (22%, 2/9) who were *P. vivax* positive in the first survey and sampled again in the second survey remained *P. vivax* positive; 12 (4.2%, 12/285) acquired a *P. vivax* infection.

**Table 2:** Overview of submicroscopic *Plasmodium* parasites and gametocytes prevalence

Index	First survey		Second survey	
	*P. falciparum*	*P. vivax*	*P. falciparum*	*P. vivax*
Overall parasite prevalence, % (n/N)	9.4 (52/551)		16.7 (49/294)	
Parasite prevalence, % (n/N)	7.8 (43/551)	2.4 (13/551)	12.9 (38/294)	4.8 (14/294)
Overall gametocyte prevalence, % (n/N)	1.3 (7/551)	2.0 (11/551)	2.0 (6/294)	4.1 (12/294)
Gametocyte prevalence among parasite positive individuals, % (n/N)	9.3 (4/43)	69.2 (9/13)	10.5 (4/38)	57.1 (8/14)
Mixed species infections among parasite positive individuals, % (n/N)	7.1 (4/56)		5.8 (3/52)	

**Fig. 2:**
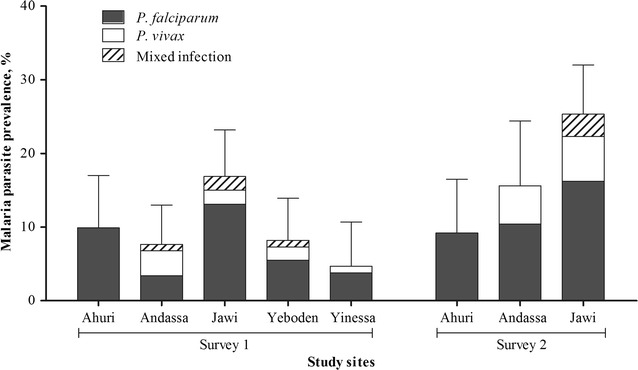
Prevalence of submicroscopic *P. falciparum* and *P. vivax* malaria carriage by quantitative PCR. Prevalence of submicroscopic carriage of *P. falciparum* (*gray bars*), *P. vivax* (*colorless bars*) and mixed infections (*hatched bars*) among children from the five schools in the first survey and three schools from the second survey is presented. *Error bars* indicate the upper limit of the 95% confidence interval

Jawi, the study site at the lowest altitude (1218 metres above sea level), had the highest prevalence of *Plasmodium* infections; 16.8% (18/107) and 25.0% (25/100) during the first and second surveys, respectively (Fig. 2). While *P. falciparum* and *P. vivax* infections were detected in Andassa, Jawi, Yeboden and Yinessa, only *P. falciparum* infections were found in Ahuri (2010 metres above sea level), where the prevalence of *P. falciparum* infection was 9.9% (11/111) during the first and 8.2% (8/98) during the second survey.

Gametocyte carriage was tested for all qPCR parasite positive and negative individuals. Of all samples from the first and second survey, 1.3% (7/551) and 2.0% (6/294) were *P. falciparum* gametocyte positive. Among *P. falciparum* qPCR positive individuals, *P. falciparum* gametocyte prevalence by (RNA-based) qRT-PCR was 9.3% (4/43) in the first and 10.8% (4/37) in the second survey (Table 2). Of all samples from the first and second survey, 2.0% (11/551) and 4.1% (12/294) were *P. vivax* gametocyte positive. Among *P. vivax* qPCR positive individuals, *P. vivax* gametocyte prevalence was 69.2% (9/13) in the first and 57.1% (8/14) in the second survey (Table 2).

### Parasite densities in relation to detectability by microscopy and gametocyte densities

**Fig. 3:**
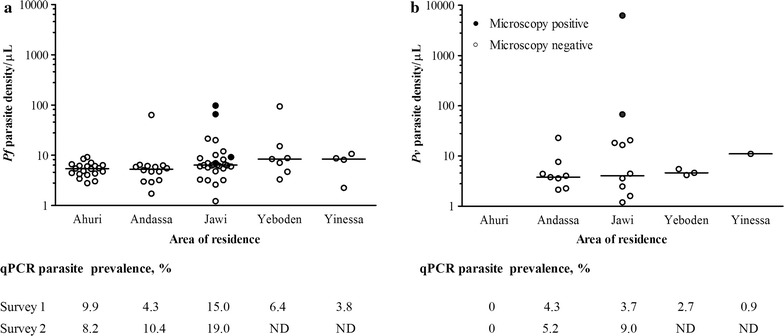
Parasite density of asymptomatic infections. Submicroscopic parasite densities as measured by 18S based qPCR for *P. falciparum* (**a**) and *P. vivax* (**b**) in microscopically positive (*black circles*) and only submicroscopic (*colorless*) asymptomatic malaria parasite carriers. Presented in the Y-axis is the Log_10_ transformed parasite density per µL of blood from the two surveys in school children from five schools (X-axis). The lines refer to the median parasite density from children in the specific school. *Numbers* below the figure indicate the qPCR parasite prevalence during the two surveys in the respective school. *ND* not determined

**Fig. 4:**
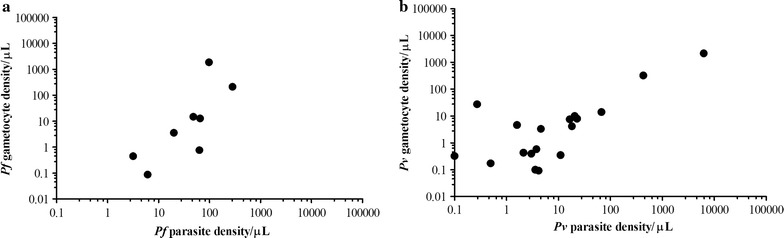
Association between parasite density and gametocyte density. The association between *P. falciparum* total parasite density versus gametocyte density (**a**) and *P. vivax* total parasite density versus gametocyte density (**b**). *P. falciparum* qRT-PCR gametocyte density was positively associated with *P. falciparum* qPCR parasite density (Spearman correlation coefficient 0.83, p = 0.010). *P. vivax* qRT-PCR gametocyte density was positively associated with *P. vivax* qPCR parasite density (Spearman correlation coefficient 0.58, p = 0.010)

Densities of *P. falciparum* and *P. vivax* infections were commonly low (Fig. 3). The 4 samples, that had microscopically detectable *P. falciparum* parasites showed higher median parasite density by qPCR (median density 326.1 parasites/μL; IQR 227.3–759.6,) compared to the 81 samples that were microscopy negative (median density 24.1 parasites/μL; IQR 17.7–32.4, p = 0.001). Similarly, the two samples, that had microscopically detectable *P. vivax* parasites also had higher parasite density by qPCR (density 56.0 and 8627.4 parasites/μL) compared to the 26 samples that were microscopy negative (median density 15.6 parasites/μL; IQR 8.5–30.5, p = 0.03). Estimated gametocyte densities were low for both *P. falciparum* (median 10.4 gametocytes/μL; IQR 3.1–51.2) and especially *P. vivax* (median 2.3 gametocytes/μL; IQR 0.7–32.0). *P. falciparum* qRT-PCR gametocyte density was positively associated with *P. falciparum* qPCR parasite density (Fig. 4; spearman correlation coefficient 0.83, p = 0.010). Similarly, *P. vivax* qRT-PCR gametocyte density was positively associated with *P. vivax* qPCR parasite density (Fig. 4; spearman correlation coefficient 0.58, p = 0.010).

### Seroprevalence and dynamics of antibody titres to malaria antigens

**Fig. 5:**
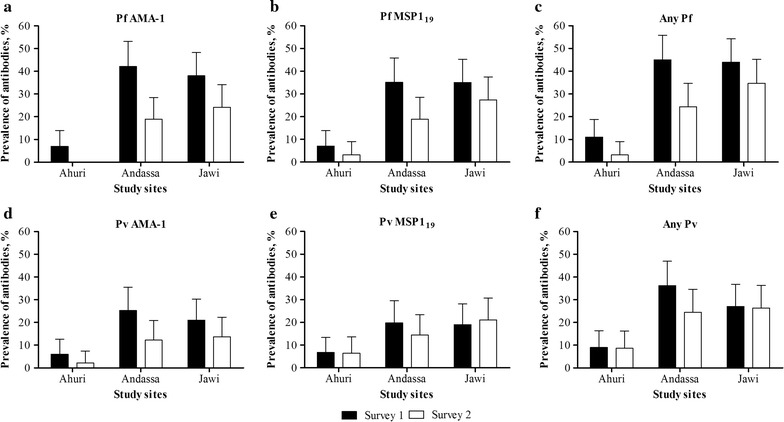
Prevalence of antibodies generated to *P. falciparum* and *P. vivax* AMA-1 and MSP-1_19_ in the study sites. The seroprevalence, positivity to antibodies generated against the *P. falciparum* AMA-1 (**a**) and MSP-1_19_ (**b**), the *P. vivax* AMA-1 (**d**) and MSP-1_19_ (**e**) during the first survey in June 2015 (*black bars*) and the second survey in November 2015 (*colorless bars*). Any Pf (**c**) and Any Pv (**d**) refers to positivity to antibodies to either of the two *P. falciparum* (**c**) or *P. vivax* (**d**) antigens. The three schools that were surveyed for the two rounds are presented in the X axis

**Fig. 6:**
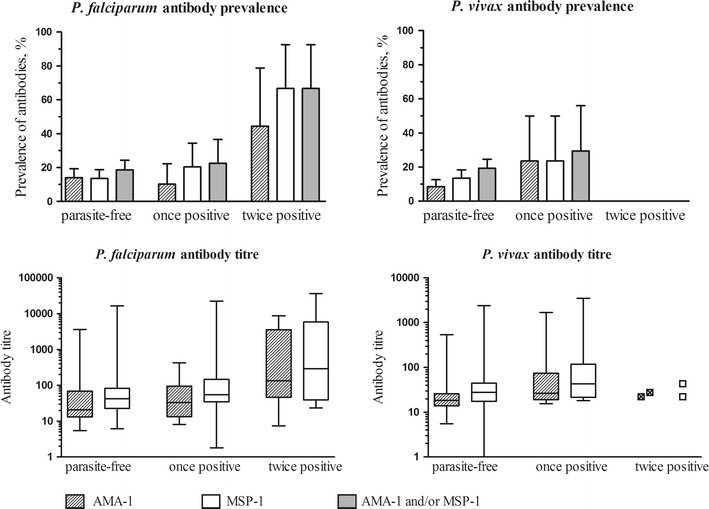
Dynamics of antibody responses. Antibody prevalence (*top*) and titre (*bottom*) for *P. falciparum* and *P. vivax* antigens in relation to qPCR detected parasite prevalence in neither of the surveys (parasite-free), in just one of the surveys (once positive) or in both surveys (twice positive). Antibody titre is presented for all individuals, including individuals whose ELISA optical density was below the threshold for positivity. The two individuals who were *P. vivax* positive in both surveys had optical density values for *P. vivax* AMA-1 and MSP-1_19_ that were below the threshold for positivity and are therefore not presented in the antibody prevalence plot

The prevalence of antibody responses to *P. falciparum* and *P. vivax* antigens was assessed as a more sensitive indicator of malaria exposure than current parasite carriage by qPCR. Seroprevalence to *P. falciparum* and *P. vivax* AMA-1 and MSP-1_19_ was strongly dependent on site (Fig. 5). The prevalence of antibodies to *P. falciparum* AMA-1 and/or MSP-1_19_ in the first survey was 11.0% (11/100) in Ahuri, 45.1% (41/91) in Andassa and 44.0% (44/100) in Jawi. In the second survey, 3.2% (3/95), 24.4% (22/90), and 34.7% (33/95) prevalence was detected, respectively. The prevalence of antibodies to *P. vivax* AMA-1 and/or MSP-1_19_ in the first survey was 9.0% (9/100) in Ahuri, 36.3% (33/91) in Andassa and 27.0% (27/100) in Jawi. In the second survey, the prevalence of any *P. vivax* antibodies was 8.5% (8/94), 24.4% (22/90), and 26.3% (25/95), respectively. After adjustment for study site, the prevalence of antibodies against either *P. falciparum* antigen was strongly associated with age in both the first (odds ratio for each year increase in age 1.29, 95% CI 1.14–1.46, p < 0.001) and second survey (OR 1.50, 95% CI 1.26–1.79, p < 0.001). For *P. vivax* antibodies, there was no statistically significant association with age (p ≥ 0.19). The prevalence and titer of *P. falciparum* antibodies was associated with parasite carriage by qPCR. Individuals who were parasite positive by qPCR in both surveys (n = 9) had a borderline significantly higher odds of being positive to either *P. falciparum* antigen in the second survey (OR 4.36, 95% CI 0.86–22.08, p = 0.076) compared to children who were parasite-negative in both surveys (n = 235), after adjustment for age and site. Individuals who were parasite positive in one but not both surveys (n = 50) had no significantly higher odds of being *P. falciparum* antibody positive in the second survey (OR 1.03, 95% CI 0.44–2.45, p = 0.94) compared to children who were parasite-negative in both surveys, after adjustment for age and site (Fig. 6). The titer of antibodies to *P. falciparum* AMA-1 (p < 0.001) and MSP-1_19_ (p < 0.001) in the second survey was also higher in individuals who were parasite positive by qPCR in both surveys compared to those remaining parasite-free, after adjustment for age and study site (Fig. 6). Individuals who were parasite positive in one but not both surveys did not have statistically significant higher antibody titers to *P. falciparum* MSP-1_19_ (p = 0.35) or AMA-1 (p = 0.93) compared to individuals who remained parasite-free. No associations between qPCR parasite carriage and antibody responses to *P. vivax* antigens were observed (Fig. 6).

Gametocyte carriage is essential for onward transmission to mosquitoes and associated with total parasite biomass [11]. At the end of follow-up (second survey), the prevalence of antibody responses to either of the *P. falciparum* antigens was 75.0% (9/12) for individuals who had *P. falciparum* gametocytes detected by qRT-PCR during the study period compared to 18.3% (49/268) for non-carriers (p < 0.001). The prevalence of antibody responses to either of the *P. vivax* antigens was 31.3% (5/16) for individuals who had *P. vivax* gametocytes detected by qRT-PCR during the study period compared to 19.0% (50/263) for non-carriers (p = 0.23).

## Discussion

The current study adds to the available evidence on the wide-scale presence of submicroscopic parasitaemia. This study quantified submicroscopic parasites and concurrent gametocytes using highly sensitive molecular assays in asymptomatic schoolchildren. Whilst only 6 infections were detected by microscopy in 881 slides, 107 of 845 blood samples were parasite positive by qPCR. A higher prevalence of malaria infections by molecular methods is commonly reported and the proportion of infections that is submicroscopic is highest in low-endemic settings [2, 3]. Nevertheless, the 18-fold higher parasite prevalence observed by qPCR compared to microscopy is remarkable [3]. Median densities were <10 parasites/μL for both *P. falciparum* and *P. vivax*, below the density of 50–100 parasites/μL that is detectable by routine microscopy [32]. Expert microscopy may detect lower parasite densities but even expert microscopy may only have a sensitivity of ~29% for detecting parasite densities in the range of 1–10 parasites/μL [33].

The findings further demonstrated that approximately 10% of all *P. falciparum* infections and 60% of all *P. vivax* infections had concurrent detectable low-density gametocytaemia [9, 11]. For both *Plasmodium* species gametocyte densities were positively associated with total parasite densities [11]. In *P. falciparum* gametocytes typically comprise <5% of the total parasite biomass. With the very low parasite densities that were detected in this study, it is conceivable that concurrent gametocyte densities were too low to be detected by qRT-PCR. On the other hand, the finding that some individuals were gametocyte positive but negative for parasites by qPCR is likely to reflect the difference in sensitivity between DNA- and RNA-based assays [4] and illustrates that some infections could have been missed by qPCR [5]. This might partly explain infection of mosquitoes even when no parasites were detected by molecular methods [34]. Higher blood volume PCR [5] or more sensitive PCR targets [35] may reduce the proportion of false-negative PCR samples although it is likely that a fraction of infections will remain undetected with currently available diagnostics [5].

The current study identified heterogeneous transmission of *Plasmodium* species in the five study sites. At four sites both *P. falciparum* and *P. vivax* were prevalent; the former was the dominant species. In one highland site, Ahuri at 2010 metres above sea level, only *P. falciparum* infections were detected. Although parasite prevalence by qPCR was relatively low in Ahuri, it remains to be demonstrated whether infections were acquired locally. The finding may reflect a shift in altitude in *P. falciparum* distribution. In warmer years, an increase in the altitude at which malaria is observed has been reported for Ethiopia and Colombia [36]. The current findings may also be influenced by climatic changes, including the El Ǹino phenomenon that occurred during the study year and resulted in intermittent rain and increased temperature across the region. Intriguingly, antibodies to *P. vivax* AMA-1 and MSP-1_19_ were detected in approximately 9% of children in Ahuri. This may reflect past exposure to *P. vivax* [31], exposure outside the area of residence, *P. vivax* exposure that is too infrequent to be detected in parasitological surveys [17], or cross-reactivity of antibodies acquired following *P. falciparum* exposure. For *P. falciparum*, antibody responses were associated with *P. falciparum* infections measured by qPCR. Individuals who were parasite positive in both surveys had higher prevalence and density of *P. falciparum* antibodies [37]. It is unclear whether this reflects boosting of antibody responses by circulating low densities of parasite antigens [38] or the fact that current infections are strongly associated with cumulative past malaria exposure [39]. Parasite prevalence and the incidence of new infections in the study populations was too low for meaningful assessments of serological markers of malaria exposure as reliable indicator of recent malaria exposure or explore the longevity of antibody responses in relation to time since last infection. However, the current findings of an association between antibody prevalence and qPCR-detected infections and qRT-PCR detected gametocyte carriage, suggest that serological markers might be useful to identify *P. falciparum* exposed individuals or populations whose infections are undetectable by microscopy but may contribute to onward transmission. It is currently unclear why this association was not observed for *P. vivax* and more detailed longitudinal studies are required to robustly demonstrate the ability of serological markers of malaria exposure to detect (recent or current) malaria infections. Recently described serological markers of recent malaria infection [40] have the potential to perform considerably better in this respect since antibodies to AMA-1 and MSP-1_19_ have a long half-life [29] and may be less informative to indicate ongoing malaria transmission in settings where transmission has declined. The age-range of the current study population was too narrow to generate a full age-seroprevalence curve that may demonstrate whether recent changes in malaria exposure have occurred [31, 41]. The association between antibody responses to *P. falciparum* blood stage antigens and *P. falciparum* gametocyte carriage is plausibly a consequence of a larger total parasite biomass in gametocyte carriers [11] that forms a stimulus for antibody production.

The relevance of the low-density parasite reservoir that was described in the current study remains to be established. Whilst chronic low-density infections may have clinical consequences [6], most attention goes to their possible role in the human infectious reservoir for malaria [10, 13]. The current study demonstrates that a proportion of the low-density infections had detectable gametocyte densities. For *P. falciparum* this proportion was only 10% which is lower than commonly reported in parasite carriers detected in cross-sectional or clinical surveys [9]. It may be argued that these infections may produce detectable gametocytes at a later stage, but the dynamics of gametocyte production and infectivity of submicroscopic infections is currently unknown. By comparison, the prevalence of gametocytes in *P. vivax* infections was higher, as has been described before, and may reflect the more rapid production and maturation of *P. vivax* gametocytes [9, 11, 42]. Efforts are currently ongoing to establish mosquito feeding facilities in the study area to determine the contribution of symptomatic, asymptomatic and submicroscopic infections to onward transmission to mosquitoes.

## Conclusions

The current findings demonstrate a considerable pool of low density infections in schoolchildren in five low endemic sites in Ethiopia that are potentially to be included in malaria elimination initiatives by the Ethiopian Ministry of Health. Serological markers may play a role in identifying individuals or populations who are disproportionately exposed to malaria and may be preferentially targeted with interventions.
